# Quality of online self-management resources for adults living with primary brain cancer, and their carers: a systematic environmental scan

**DOI:** 10.1186/s12904-021-00715-4

**Published:** 2021-01-23

**Authors:** Isabelle Schaefer, Nicole Heneka, Tim Luckett, Meera R. Agar, Suzanne K. Chambers, David C. Currow, Georgia Halkett, Domenica Disalvo, Ingrid Amgarth-Duff, Cleola Anderiesz, Jane L. Phillips

**Affiliations:** 1 Improving Palliative, Aged and Chronic Care through Clinical Research and Translation (IMPACCT), Faculty of Health, University of Technology Sydney, Sydney, New South Wales Australia; 2 Faculty of Health, University of Technology Sydney, Sydney, New South Wales Australia; 3 School of Nursing, Midwifery and Paramedicine, Psychology Building, Curtin University, Perth, Western Australia Australia; 4 Australian Brain Cancer Mission, Cancer Australia, Sydney, New South Wales Australia

**Keywords:** Brain neoplasms, Consumer health information, Self-management, Cancer, Online

## Abstract

**Background:**

A primary brain cancer diagnosis is a distressing, life changing event. It adversely affects the quality of life for the person living with brain cancer and their families (‘carers’). Timely access to evidence-based information is critical to enabling people living with brain cancer, and their carers, to self-manage the devastating impacts of this disease.

**Method:**

A systematic environmental scan of web-based resources. A depersonalised search for online English-language resources published from 2009 to December 2019 and designed for adults (> 25 years of age), living with primary brain cancer, was undertaken using the Google search engine. The online information was classified according to: 1) the step on the cancer care continuum; 2) self-management domains (PRISMS taxonomy); 3) basic information disclosure (Silberg criteria); 4) independent quality verification (HonCode); 5) reliability of disease and treatment information (DISCERN Sections 1 and 2); and readability (Flesch-Kincaid reading grade).

**Results:**

A total of 119 online resources were identified, most originating in England (*n* = 49); Australia (*n* = 27); or the USA (*n =* 27). The majority of resources related to active treatment (*n* = 76), without addressing recurrence (*n* = 3), survivorship (*n* = 1) or palliative care needs (*n* = 13). Few online resources directly provided self-management advice for adults living with brain cancer or their carers. Just over a fifth (*n* = 26, 22%) were underpinned by verifiable evidence. Only one quarter of organisations producing resources were HonCode certified (*n* = 9, 24%). The median resource reliability as measured by Section 1, DISCERN tool, was 56%. A median of 8.8 years of education was required to understand these online resources.

**Conclusions:**

More targeted online information is needed to provide people affected by brain cancer with practical self-management advice. Resources need to better address patient and carer needs related to: rehabilitation, managing behavioural changes, survivorship and living with uncertainty; recurrence; and transition to palliative care. Developing online resources that don’t require a high level of literacy and/or cognition are also required.

**Supplementary Information:**

The online version contains supplementary material available at 10.1186/s12904-021-00715-4.

## Background

In 2018, 296,851 people across the world were diagnosed with brain cancer and 241,037 died as a result of this cancer [1]. Globally, little progress has been made in improving five-year survival rates for primary brain cancer (‘brain cancer’) [2]. Even in long-term survivors, cognitive and physical disability associated with brain cancer can be profound. The overall disease burden from brain cancer has been estimated as 7.7 million disability-adjusted life years [3]. As many adults are of working age, with a partner, children, other family and financial responsibilities, a brain cancer diagnosis impacts adversely on every aspect of the person’s life, as well as those of their family and carer(s) (‘carers’) [4].

The information needs of people living with brain cancer, and their carers, change according to their phase of disease [5, 6]. The Australian National Service Improvement Framework for Cancer [7] identifies key intervention points across the cancer care continuum, namely: reducing risk, finding cancer early, care between and after treatment, and end of life care. People diagnosed with high grade glioma and other cancers find it difficult to process complex prognostic information, and experience a strong and pressing need for information including treatment options, side effects and prognosis [5, 6, 8].

Much of this need for timely information is driven by the desire of people with any type of cancer to make informed decisions and to plan accordingly, but also to shape the questions to ask clinicians before and after the initial consultation [8–10]. People with high grade brain cancers require information early in the disease course to enable them and their carers to adjust to their sudden and profound reduction in independence due to: decline in cognitive function; changes in physical abilities including onset of seizures and balance problems; reduced ability to work or drive and associated loss of income, as well as the emotional toll of their diagnosis [6, 11]. Later in the cancer care continuum, people with brain cancer and their carers require access to information to help them self-manage their symptoms such as fatigue and memory deficits that can vary in severity over time, maintain emotional well-being and independently undertake activities of daily living as much as possible. Long-term brain cancer survivors and their carers need information on strategies for rehabilitation and management of long term symptoms including difficulty assimilating and remembering information [6, 12, 13]. Given the high rate of recurrence in brain cancers, information on palliation and end of life care is also critical for both people with brain cancer and their carers [13, 14].

Online searching for information is often the first step people with newly diagnosed cancer of any type independently undertake in order to start to understand the implications of their condition and/or symptoms [15]. People living with any cancer, and their carers, often rely on online resources to supplement information and advice provided by clinicians, despite the fact that online resources may contain misleading or incorrect information [16–18]. However, people living with brain cancer report difficulty in finding and comprehending online information that addresses their needs, including strategies to maintain psychosocial wellbeing and symptom management, particularly in the presence of difficulties in concentration and understanding [6]. The quality and readability of online resources is therefore critical to ensure that people with brain cancer and their carers have access to appropriate, accurate evidenced based information that is easy to comprehend and integrate.

Accessing accurate information is essential to supporting the self-management actions people take to cope with their illness [19–22]. Information that contributes to strengthening the person’s capacity to self-manage their cancer enables them to: feel more prepared for interventions such as surgery and to cope with their post-operative symptoms; feel confident to participate in medical decision-making; and promotes adherence to recommended treatments [21, 22]. People who are better informed about their disease have less anxiety and depression, and better self-management and treatment adherence [23, 24].

Upon, and immediately beyond diagnosis, the information received uniquely shapes a person’s attitudes to their condition [21]. The provision of timely information initiates an iterative process of learning and implementation that promotes feelings of empowerment and confidence [25, 26]. The benefits of online information have given rise to a variety of practical online resources available for self-management of illness, including question prompt lists, symptom diaries, exercise and diet programs, and psychotherapeutic and cognitive training resources. However, it remains unclear how many resources are available for adults affected by brain cancer, or the quality of these resources.

### Aim

To appraise the content, reliability and readability of the available online self-management resources for adults living with primary brain cancer, and their carers.

## Methods

### Design

An environmental scan, conducted and reported in accordance with the PRISMA Statement [27].

### Inclusion criteria

To be included, online consumer resources (‘resources’) needed to be available free of charge in English and provide text-based advice that an adult (aged > 25 years) living with brain cancer or a caregiver, (‘consumer’) might use to inform self-management across the brain cancer care continuum [7]. Self-management was defined in terms of the 14 domains identified by the Practical Reviews in Self-Management Support (PRISMS) taxonomy [28]. Included resources needed to contain at least one PRISMS component addressing practical self-management (components A2-A14, see Table 2). Resources that only comprised information about disease (component A1), and no other PRISMS components, were excluded to maintain focus on practical self-management advice. Resources did not need to specify phase of disease management or age, but those specific to non-adult populations managing brain cancer (e.g., paediatric, adolescent and young adult (aged 15–24 years) were excluded, due to their specific developmental needs. Additionally, resources directly referring to cerebral metastases, or benign tumours (WHO Grade I) were excluded [29].

To improve confidence in the currency and quality, resources had to be developed from 2009 onwards, and originate from countries identified as being within the top twenty for five-year brain cancer survivorship by the *Global surveillance of trends in cancer survival 2000–14 (CONCORD-3)* [2].

### Searches

The web-based search was conducted using the Google search engine, in the Google Chrome web browser. Searches were depersonalised by adding “&pws = 0” to the URL to prevent tailoring of results to user or computer. The search strategy incorporated the name of each country, and variations of ‘brain cancer’ and specific tumour types as identified in a systematic review of brain cancer research [30].

#### Data collection and analysis

Data collection and analysis was undertaken by three authors (IS, DS and IAD). A subset of resources were cross-checked between additional authors (TL and JLP), and discrepancies resolved by discussion with the larger author team. Data on relevant resources were extracted into an MS Excel spreadsheet to capture country of origin, organisation name, resource name and URL. The format of the resource (web page or digital document [PDF]) and organisation type (charity, government, professional, commercial) were also recorded. The focus of the information was classified according to phase of the cancer care continuum, with the addition of sub-domains specific to care of people with brain cancer identified through thematic content analysis [7]. Self-management content was categorised according to the PRISMS taxonomy [28].

The quality of resources was appraised using the following quality assessments.

- *Silberg Criteria* [31]: The availability of information needed for a consumer to judge the quality of a resource were rated using the four criteria developed for online heath information: date of publication, authorship, reference to evidence and disclosure of competing interests. These criteria represent the minimum core standards that should be met by online health resources to enable consumers to assess whether a resource is reliable and accurate [26].
- *HonCode Certification* [32]: Provided by the Health on the Net Foundation, websites are evaluated by medical experts using eight principles: authority, complementarity, confidentiality, attribution, justifiability, transparency, financial disclosure and advertisement policy. Certification indicates that a website provides detailed information about the developing organisation, and that health information is complete, balanced and transparent. This was measured using the HonCode toolbar [33].
- *DISCERN tool* [34]: To assess the quality of each resource in detail, sections one and two of the DISCERN tool were used. The DISCERN tool is a three-part tool designed for consumers to rate the reliability of written health information [34]. This tool was modified to include a three-point scale (0 = lowest; 1 = partial; 2 = highest) after finding seven-points to be too fine grained to reliably rate. Resources were rated by clarity, transparency, bias, scope and referral to further information (Section 1); and balance/thoroughness of treatment information (Section 2). Section 3, a global rating score, was not included so that all resources were rated in the same way.
- *Flesch-Kincaid reading grade tool* [35]: This tool uses a formula to rate the complexity of text and estimates the grade/year of education (United States) required to understand the text for ease of interpretation [35]. Text from resources was copied into a MS Word document. Any complex elements such as web URLs, phone numbers or addresses were removed. Text from bullet points was retained, but converted into single sentences. The text was then analysed by an online calculator [36].

When reporting overall quality, the DISCERN Section 1 score rating clarity, transparency, bias and use of references to evidence was used [34]. This was designed to enable all resources to be graded consistently by the same overall measure. Resources were divided into quartiles to facilitate identification of the highest ranking resources.

## Results

**Fig. 1:**
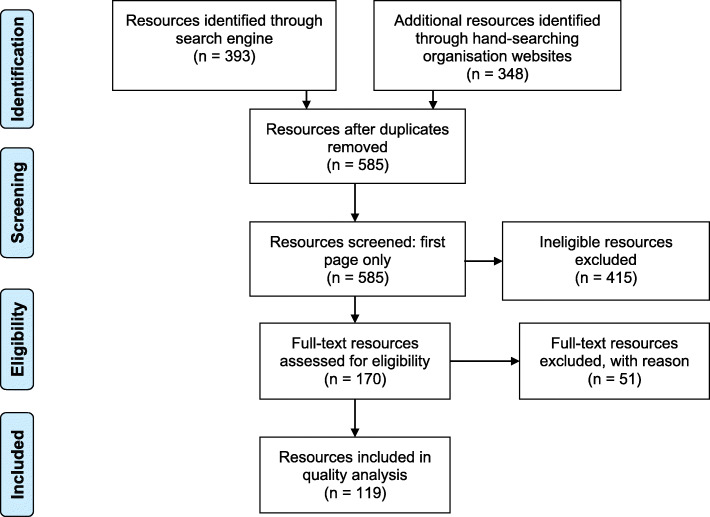
PRISMA flowchart showing screening and eligibility assessment

A total of 741 resources were identified during the search, of which 119 met the inclusion criteria (Refer Fig. 1). A summary of data analysis is provided in Additional file 1.

Resources for consumers about self-management for people living with brain cancer and their carers were developed in England (*n* = 49), Australia (*n* = 27), the US (*n =* 27), Canada (*n* = 11), Ireland (*n =* 3) and Scotland (*n =* 2).

### Cancer care continuum

The majority of resources available for adults living with brain cancer, and their carers, comprised topics relating to ‘active treatment’ (*n* = 76) and ‘care between and after treatment’ (*n* = 121) (Refer Table 1). Palliative and end-of-life care (*n* = 13) and assessment and management of recurrent disease (*n =* 3) had the smallest number of resources across the cancer care continuum, while long-term survivorship (*n =* 1) considerations were the least described sub-domains.

**Table 1:** Resources mapped to cancer care continuum by subdomain (*N* = 119)

Phases of Cancer Continuum [7]	Number of references to subdomains (***n*** = 119)^**a**^
**Find cancer early**	**43**
**Active treatment**	**76**
*Surgery*	19
*Systemic therapy*	16
*Radiotherapy*	19
*Supportive care for side effects during treatment*	18
*Alternative and Complementary treatments*	4
**Care between and after treatment**	**131**
*Self-efficacy*	15
*Carer Support*	12
*Care Coordination*	5
*Surveillance*	3
*Rehabilitation*	36
*Long term survivorship*	1
*Management of long-term treatment side effects*	30
*Psychosocial care*	29
**Recurrent Disease**	**3**
**Palliative and End-of-life care**	**13**

^a^Total number of discrete resources differs from column total where resources were relevant across more than one point in the cancer care continuum

### Prisms

Most resources (*n* = 80, 60%) for adults with brain cancer and their carers included basic information about the nature and treatment of the disease (Refer Table 2). Just under half (*n* = 62, 47%) included lifestyle advice and support and approximately one third (*n* = 46, 35%) included information about available resources. No resources provided information on accessing equipment to support daily life, and few resources promoted regular review (*n* = 1, 1%) or symptom tracking with clinical review (*n* = 2, 2%).

**Table 2:** Categorisation of resources by PRISMS component (*N* = 119)

PRISMS component	Total number of discrete resources (***N =*** 119)^**a**^	
	n (%)	
A1. Information about condition and /or its management	66	(50%)
A2. Information about available resources	34	(26%)
A3. Provision of/agreement on specific clinical action plans and/or rescue medication	7	(5.3%)
A4. Regular clinical review	1	(0.8%)
A5. Monitoring of condition with feedback	2	(1.5%)
A6. Practical support with adherence (medication or behavioural)	9	(6.8%)
A7. Provision of assistive equipment	0	(0%)
A8. Provision of easy access to advice or support when needed	19	(14%)
A9. Training/rehearsal to communicate with health-care professionals	24	(18%)
A10. Training/ rehearsal for everyday activities	10	(7.5%)
A11. Training/ rehearsal for practical self-management activities	19	(14%)
A12. Training/ rehearsal for psychological strategies	42	(32%)
A13. Social support	22	(17%)
A14. Lifestyle advice and support	58	(44%)

^a^Total number of discrete resources differs from column totals where resources addressed more than one PRISMS component

### Silberg criteria scores

Half (*n* = 67, 50%) of the resources fulfilled more than half (≥ 3) of Silberg’s criteria, but only one tenth (*n =* 14, 11%) fulfilled all four minimal reporting criteria for online health information. All resources recorded the funding or developing organisation, however just under three-quarters (*n* = 95, 71%) included details about when the resource was published or last updated. Only a third (*n* = 48, 36%) included information about authorship and one quarter (*n* = 37, 28%) referenced reliable evidence used to develop resource.

### HonCode certification

Only one quarter (*n* = 9, 24%) of the organisations (*n* = 32) that developed the identified resources were HonCode certified.

### DISCERN sections 1 and 2

#### Section 1

**Fig. 2:**
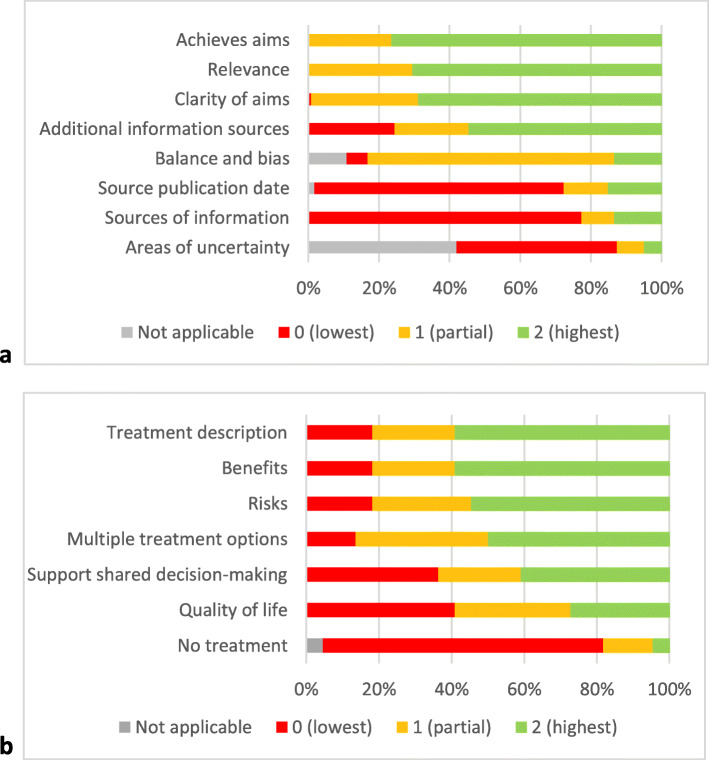
Quality analysis of resources using DISCERN Section 1 (Panel **a**, *n* = 119), and DISCERN Section 2 (Panel **b**, *n* = 22)

Median percentage score resource reliability was 56% (range 25–100%) (Refer Fig. 2). Sixty eight percent of the identified resources had a clearly defined purpose. The resources often contained information about additional support services or information providers relevant to people living with brain cancer and their carers. Resources often did not cite reliable health information to support content (*n* = 92, 77%), or when cited evidence was published (*n* = 84, 71%). Eighty three percent of resources were rated as mostly or entirely balanced and unbiased (*n* = 99). However, resources rarely referred to areas of uncertainty such as predicting personal surgical outcomes or treatment responses (*n* = 15, 22%).

#### Section 2

Almost one fifth (*n* = 22, 18%) of all resources included information about brain cancer treatments. The median DISCERN Section 2 score for the quality and bias of treatment information was 57% (range 14–93%). Approximately one third of the resources (*n* = 6, 36%) rated greater than 70% for quality of treatment information. A small number of resources (*n* = 4, 18%) rated less than 30% for reliability.

Resources often gave a basic description of brain cancer treatment(s) and its benefits (*n* = 18, 82%). Only one resource mentioned the option of not receiving treatment for brain cancer. Description of impact on quality of life (*n* = 13, 59%) and support for shared decision-making (*n* = 14, 64%) were included less frequently.

### Flesch-Kincaid reading grade level

The median score for years of education required to understand resources was grade 8.8 (range 4.1–15).

### Analysis of top quartile resources

Resources in the top quartile scored 69% or above in DISCERN Section 1 (Additional file 1). Number of PRISMS categories included in resource, Silberg criteria rating and reading grade required were approximately equal when comparing the top quartile resources to resources in the lower quartiles (Refer Table 3). Two thirds (*n* = 20, 66%) of the top quartile resources were published digital documents (portable document format, [PDF]). Compared to the three lower quartiles, the resources in the top quartile were more likely to include a publication date (93% vs 63%) and verifiable evidence to support content (73% vs 3%).

**Table 3:** Comparison of resources by quartile (*N* = 119)

Resources	Top quartile (***n*** = 31)		Lower three quartiles (***n*** = 88)	
**PRISMS** (median)	2.5		3	
**Silberg Criteria** (median)	75%		50%	
*Date (n, %)*	28	(93%)	55	(63%)
*Authorship (n, %)*	13	(43%)	33	(37%)
*References (n, %)*	22	(73%)	3	(3%)
*Disclosure (n, %)*	30	(100%)	88	(100%)
**Reading Grade** (median)	8.8		8.75	
**Document type**				
*Webpage (n, %)*	10 (33%)		44 (50%)	
*Digital Document (n, %)*	20 (66%)		44 (50%)	

## Discussion

This environmental scan found that self-management resources for people living with brain cancer often did not adhere to minimum reporting standards for online health information [31]. As a result, people living with brain cancer, and their carers, may find it difficult to judge quality and reliability, and the existing online information may not address their evolving needs throughout the different phases of their disease.

Content did not equally cover all phases of the cancer care continuum, and contained very little practical self-management advice to assist people living with brain cancer to play an active role in their care. In addition this environmental scan found that few resources were underpinned by verifiable evidence, or tailored to address the needs of people with cognitive impairment from culturally and linguistically diverse backgrounds or First Nations people.

### Distribution across cancer care continuum

The identified self-management resources did not equally address the whole brain cancer care continuum. While desire for information is highest at the time of diagnosis, information needs persist as people with cancer transition between management phases, undertake new treatments or therapies, or disease management changes [5]. However, resources identified in this study most often addressed issues in the early to intermediate phases of brain cancer management; encompassing diagnosis, treatment choices, decision-making, care coordination and side effect management. In comparison, resources that spoke to the unique needs of brain cancer survivors were lacking, such as specific post-treatment self-management strategies for people with no sights of active disease. To some extent, the volume of resources surrounding diagnosis and early treatment decision-making for people living with brain cancer is commensurate with evidence that this is the most intense period of information seeking for many patients and their carers [5]. This may be particularly evident in aggressive brain cancer, where there is less time to seek information between diagnosis and active management due to the urgent need to commence treatment [25]. However, cancer survivors, regardless of cancer type, report having significant unmet information needs once their treatment has been completed [5, 37, 38].

There are few online resources that address brain cancer recurrence, despite the high risk of recurrence for all brain cancers [14]. People with cancer, and their carers, report that their greatest information need during recurrence was clear, honest communication about prognosis, yet this information is not readily available online for people living with brain cancer [39, 40]. Similarly, there are very few references to palliative and end of life care in any of the resources, which is a significant gap given the poor prognosis of people with brain cancer [2, 41–50]. A recent study in the US demonstrated that people living with brain cancer often express fear when discussing palliative care and associate it with end of life [51]. Once they had information about the role of palliative care, people living with brain cancer believed that a palliative approach would enhance their mental wellbeing, and wished for information about palliative care earlier in disease course [51]. They also suggested that they would be more open to palliative care if it was not framed as an end of life intervention [51]. It is highly likely that people living with brain cancer, and their carers, would benefit from early access to information about palliative care so that they can make more informed choices about their treatment and symptom management earlier in their illness trajectory.

### Lack of practical self-management advice

People who have recently completed their primary cancer treatment are often anxious as they transition away from an acute care model into a new phase of living with their cancer, and many experience difficulty when trying to self-manage side effects and recovery from illness [38]. While disease information is a factor in supporting self-management of chronic illnesses, people living with brain cancer also require practical advice to assist them coordinate their treatment, symptom management and activities of daily living [28]. Carers also have a strong need for self-management information as cognitive or memory dysfunction can result in people being with brain cancer being unable to undertake complex tasks such as medication management, and experience behavioural change, which is challenging to manage in the home [6]. Unfortunately, very few resources provide practical self-management advice, such as how to effectively manage: cognitive and behavioural changes, treatment side-effects, rehabilitation; and assess capacity to continue to work and/or drive; as opposed to basic factual disease information for this population.

The paucity of self-management guidance for people living with brain cancer on how to live with cognitive and physical disabilities, disease and treatment side-effects and the importance of rehabilitation, and information for carers on how to assist them, is a missed opportunity. Access to reliable information is critical to enabling people living with brain cancer to make independent, informed decisions that will improve their quality of life and ensure that they feel empowered to engage in medical decision-making [17]. Resources that enable people living with brain cancer to engage in rehabilitation at home are of particular importance given the documented reluctance of some clinicians and rehabilitation services to offer people living with brain cancer access to rehabilitation [52]. Clinicians’ reluctance to offer rehabilitation to people living with brain cancer may stem from a misconception that they are not well enough to participate, have a poor prognosis and/or that rehabilitation has little role in maintaining quality of life for this population [52].

The availability of high quality, accessible and detailed information for carers is also vital to provide support as the person with brain cancer becomes more dependent on their care due to side effects from illness and treatment [6, 11, 13]. High quality guides for carers were available online, and described useful and practical management and planning strategies such as planning care, financial preparations and self-care while caring for someone else. However, these could be improved by reducing the reading grade required.

Given the often fragmented nature of brain cancer care across disciplines and care settings during the acute phase, people living with brain cancer, and their carers, are often required to coordinate their own care [53]. The rapid change in circumstances that accompanies brain cancer diagnosis also creates erosion of potentially supportive social networks [54]. This may result in people living with brain cancer, and their carers, feeling less able to initiate new self-management practices after treatment through lack of stable clinical and social support [53].

There is significant opportunity to provide information on the value of low cost, self-determined, home-based interventions such as: exercise programs to benefit physical health and manage fatigue [55], mindfulness to improve cognitive function [56] and pain management that are specific to or can be tailored to people with brain cancer [57]. This would be of particular use for people living with brain cancer that experience seizures, where caregivers report fear of seizures makes leaving the house difficult [58]. Information about these programs in online resources presents significant advantages to people living with brain cancer. These self-management strategies and programs could be readily integrated with routine clinical care to enhance patient outcomes, be home-based in accordance with the preference of many adults with glioma [59] and enable a feeling of greater control [60]. People with cancer that engage in self-management activities outside of the healthcare context can demonstrate gains in: physical function [55], cognition [61], mental health [62] and pain management [57]. The development of appropriate resources that are accessible and practical has the potential to be of great benefit to people living with brain cancer as well as their carers.

### Quality of resources

Similar to other assessments of online information for various cancers, the quality of resources for brain cancer is highly variable [63]. In this study, resources in the top quartile for quality were more likely to contain information that may assist people living with brain cancer, and their carers, to gauge reliability, such as the date of publication and the inclusion of references to information that support the advice or information provided. Conversely, many resources from the lower three quartiles did not. Interestingly, resources in the top quartile were more likely to be published as a digital document (PDF) than as a webpage. No conclusions can be drawn from this data, but this may be worthy of further examination to determine whether this may be a useful indicator of quality to people with brain cancer. Many resources also exceeded the recommended reading grade level, reducing the accessibility of self-management advice and information about disease.

Consumers of online health information are vulnerable to inaccurate and unreliable information [31]. People living with brain cancer, and their carers, need to be able to readily discern between poor and high quality information or self-management advice, which relies on robust health literacy and evaluation skills [64]. Given people with poor health literacy can demonstrate limited understanding and application of tailored self-management advice given directly by clinicians, it follows that this may be compounded when they are required to use: self-initiated strategies to search for specific information; critical thinking to differentiate quality resources; and depth of understanding to assimilate and act on advice [65]. Consumers seeking information referring to complementary and alternative therapies are at particular risk of accessing unreliable information, as these therapies may not be based on evidence. In part, this gap is recognised by non-government organisations providing information for people living with brain cancer, and their carers, that includes guidance for consumers about how to make informed choices about unproven treatments.

The vulnerability of those with poor health literacy to unreliable online self-management advice, or information, is compounded by the short period of time between brain cancer diagnosis and treatment characteristic of serious and progressive illness, and the urgent need for information early in disease course [5, 25]. Cognitive dysfunction in the form of impaired reasoning, processing and memory may further compound this issue for people living with brain cancer by reducing understanding of disease and therefore participation in care [66]. Omission of details of a self-management resource such as date of publication and use of verifiable evidence sources means people living with brain cancer, and their carers, may not be able to easily judge whether a self-management resource is trustworthy.

Substantive information about advance care planning and appointing guardians or medical decision-makers was rarely included in brain cancer specific resources. This deficit in information is particularly important for people with brain cancer, especially those with high-grade disease who have a very poor prognosis, and it has been shown that end of life care discussions occur late in disease course when the patient is most dependent [2, 67]. Some carer resources made reference to these topics, but there appears to be a need for high quality resources aimed specifically at people with brain cancer to be developed, especially for those with reduced cognition.

Practical self-management resources for people living with brain cancer, including information about disease, should be clearly supported by reliable evidence to enable them to rapidly discern and adopt high quality resources, and be regularly updated to reflect current evidence. Co-designing new brain cancer resource with those affected will ensure that future resources address this populations specific information needs across the brain cancer continuum.

### Strengths and limitations

This study encompassed a detailed, systematic search of online health information that emulated the search habits of consumers. Consumer-friendly search terms were used to search the market-leader in online search engines to simulate a search by a lay-person.

Limitations of this study include that only one search engine and language were used. While resources developed before 2009 were excluded, many websites did not include dates, bringing into question the currency of information in these cases. It should also be noted that a study of this kind can only provide a ‘snapshot’ of the dynamic online environment that quickly evolves. By limiting to text-based information, we overlooked other kinds of online resources including ‘social web’ opportunities such as peer support, and mHealth apps.

### Implications for research and practice

The development of evidence-based resources in partnership with consumers, clinicians and researchers would result in relevant, accessible content that is evidence-based. Clinicians need to direct people living with brain cancer, and their carers to the best accessible, evidence-based resources. Having ready access to high quality, reliable information would enable people living with brain cancer, and their carers, to actively participate in planning and undertaking their disease management, and enhance emotional and physical wellbeing.

## Conclusion

This systematic environmental scan has demonstrated that there is a plethora of consumer resources. However, none address all of the needs of adults living with brain cancer or their carers across the cancer care continuum, very few are evidence-based and only a small number meet the recommended readability standards. Very few of the resources were designed for people with cognitive impairment or for people from culturally and linguistically diverse backgrounds or First Nations people. There is a significant gap in online resources that provide practical self-management advice, especially in relationship to: survivorship; living with uncertainty; managing behavioural changes; rehabilitation; recurrence; and transition to palliative care.

## Supplementary Information

**Additional file 1.** Quality and readability analysis of online resources for people living with brain cancer and their carers. A list of all resources captured, including country of origin; type and name of developing organisation; and quality and readability scores.

## Data Availability

The dataset supporting the conclusions of this article is included within the article and its additional file.
